# Acceptability and readiness to promote human papillomavirus vaccination at ages 9–10 years: a feasibility study among North Carolina clinics

**DOI:** 10.1186/s40814-023-01379-y

**Published:** 2023-08-31

**Authors:** Nadja A. Vielot, Robyn M. Lane, Kaitlyn Loefstedt, Jennifer L. Cunningham, Jason Everson, Eli Tiller, Sarah E. Johnson Patel, Jennifer S. Smith

**Affiliations:** 1 Department of Family Medicine, University of North Carolina at Chapel Hill, 590 Manning Drive, Chapel Hill, North Carolina 27599 USA; 2 Department of Health Behavior, University of North Carolina at Chapel Hill, Chapel Hill, North Carolina USA; 3 Piedmont Health Services, Inc, Chapel Hill, North Carolina USA; 4 Department of Epidemiology, University of North Carolina at Chapel Hill, Chapel Hill, North Carolina USA; 5 Lineberger Comprehensive Cancer Center, University of North Carolina at Chapel Hill, Chapel Hill, North Carolina USA

**Keywords:** Human papillomavirus, Vaccines, Providers, Recommendations, Adolescents, Rural, Underserved

## Abstract

While 9-valent human papillomavirus (HPV) vaccination is approved by the US Food and Drug Administration for use in adolescents as young as age 9, providers typically recommend it at ages 11–12. Studies suggest that recommending HPV vaccination at 9 or 10 years of age could increase up-to-date vaccination by age 13, which could especially benefit rural populations with reduced access to primary health care and lower HPV vaccination coverage than urban areas. This study aimed to assess the feasibility of the age-9 recommendation of HPV vaccination in rural clinics. We conducted in-depth interviews with providers and staff from two primary care clinics in central North Carolina to understand attitudes toward recommending HPV vaccination to 9- and 10-year-olds. All interviewees agreed that HPV vaccination was important for cancer prevention and should be recommended before the onset of sexual activity, agreeing that HPV vaccination could be initiated before age 11 to improve timeliness and completion of the vaccination series. However, opinions were mixed on whether HPV vaccination should be initiated as young as 9 years old. Two key informants recruited from two university-affiliated clinics described their experiences recommending HPV vaccination to 9- and 10-year-olds, including a modified vaccination schedule that promotes HPV vaccination during routine well-child visits, prior to pubertal onset, and alongside other recommended adolescent vaccines. Age-9 recommendation and administration of HPV vaccination is possible with minimal changes to current clinical practices and could increase the convenience and acceptability of HPV vaccination in under-vaccinated settings.

**Supplementary Information:**

The online version contains supplementary material available at 10.1186/s40814-023-01379-y.

## Key messages regarding feasibility

- It is unknown if clinics that provide human papillomavirus (HPV) vaccination to adolescents in rural areas of the USA are willing or able to recommend HPV vaccination starting at age 9, per US Food and Drug Administration licensure.
- In 10 interviews with clinic personnel, we determined that clinic personnel in rural, as well as urban settings, were largely willing to recommend HPV vaccination to 9- and 10-year-old patients, but some expressed hesitancy about the need to vaccinate at age 9 and the feasibility of adapting current clinic practices.
- Two key informants with experience recommending HPV vaccination at ages 9–10 years provided insight on best practices to integrate this change into clinic practice, which can be evaluated in future randomized trials.

## Background

As of 2022, 117 countries have implemented universal human papillomavirus (HPV) vaccination programs that recommend two doses of HPV vaccine to prevent up to 90% of cervical cancers and large proportions of vulvar, vaginal, anal, and head and neck cancers caused by persistent high-risk HPV infection [1–3]. In the USA, HPV vaccination has been universally recommended to 11- and 12-year-olds since 2011 [4]. However, in 2020, less than half (45.6%) of adolescents in the USA had completed the HPV vaccination series on time, defined by the Centers for Disease Control and Prevention (CDC) as before their 13th birthday [5, 6]. Furthermore, HPV vaccination rates are disproportionately lower among rural adolescents, who are up to 25% less likely than urban adolescents to initiate or complete the HPV vaccination series [7]. Compared to cities, rural areas tend to also have higher cancer incidence and mortality, including cervical cancer [8, 9]. As such, improving HPV vaccination rates is a priority in rural areas.

While the Food and Drug Administration (FDA) has approved HPV vaccination for adolescents starting at age 9 based on safety and efficacy data from clinical trials [10], recommendations about when providers should begin recommending HPV vaccination differ across leading health organizations. The CDC routinely recommends initiating HPV vaccination at ages 11–12 as part of an adolescent vaccination package with tetanus-containing (Tdap) and quadrivalent meningococcal (MenACWY) vaccines, defining “up-to-date” routine vaccination as the receipt of two doses by age 13. In contrast, the American Cancer Society and American Academy of Pediatrics have recently recommended routine initiation at age 9 [11, 12], while the CDC only allows discretional use of HPV vaccination for those ages 9–10 [13]. Studies from urban centers have shown that adolescents who initiate HPV vaccination at ages 9–10 are more likely to be fully vaccinated by age 13, compared to those who initiated at age 11 or older [14, 15]. A benefit of age-9 HPV vaccination includes allowing more time to complete the vaccination series by age 13 and before the initiation of sexual activity, which may be beneficial to adolescents who experience healthcare disruptions or reduced access to routine preventive care.

While early HPV vaccination findings are promising, questions remain about the feasibility of translating these findings into health system change, specifically in rural communities where the need is greatest. To effectively implement early HPV vaccination recommendations, clinics must be able to monitor vaccination rates of 9- and 10-year-olds, and vaccine providers must be willing to recommend and provide HPV vaccination to this younger age group. In a recent national survey, only 21% of primary care professionals reported that they routinely recommend HPV vaccination at ages 9–10; however, 61% reported being willing to start recommending HPV vaccination at age 9, with no differences in willingness between urban and rural providers [16]. Thus, an age-9 HPV vaccination strategy could possibly be feasible in rural settings where provider willingness is high.

With a rural population of approximately 2 million people [17], North Carolina (NC) offers opportunities to test HPV vaccination promotion strategies in rural populations with reduced access to cancer screening and treatment. This study aimed to assess the feasibility of age-9 recommendation of HPV vaccination in a small sample of NC clinics that provide HPV vaccination to adolescents, including one clinic that serves a largely rural county. Findings from this study will inform the design of larger and more in-depth studies to assess clinic workflow around HPV vaccination, the feasibility of recommending age-9 HPV vaccination, and the effectiveness of clinic-based interventions to recommend age-9 HPV vaccination. Implementation science approaches can then be used to adapt this promising intervention to rural areas that stand to gain the most from cervical cancer prevention efforts.

## Methods

### Study sites and participants

We actively recruited two clinics from a system of federally qualified health centers that serve patients throughout central NC (“primary clinics”). The study was reviewed and approved by health system leadership, and clinic-based leaders identified clinicians and other clinic personnel to complete interviews. Eligible clinics recommended routine HPV vaccination to adolescents ages 11–12 years, and we purposively sought out clinics that served communities with majority-rural residents. Eligible interviewees within clinics included any provider who discussed HPV vaccination with patients and caregivers, medical staff who administered HPV vaccination, and administrative staff who monitored HPV vaccination rates, conducted patient outreach for HPV vaccination, or scheduled HPV vaccination visits.

In addition, we identified providers at urban practices in a neighboring academic health system who indicated that they were already recommending and providing HPV vaccination to 9- and 10-year-old patients during a separate research study on HPV vaccination. We invited these providers to participate in post hoc in-depth key-informant interviews to describe their experiences and best practices for recommending age-9 HPV vaccination.

### Primary clinic and patient characteristics

We asked a clinic administrator or a lead provider in each primary clinic to respond to a questionnaire describing characteristics of their clinics and patients, and clinical practices around HPV vaccination. Using existing EHR queries developed and validated by clinic IT staff, we estimated the number of active patients eligible for two-dose HPV vaccination (i.e., patients ages 9–13 who completed a routine medical visit (i.e., well-child check) in the clinic in the last 18 months) and the proportion who had initiated or completed the HPV vaccination series in each clinic.

### Development of the interview guide

The research team, which included members with expertise in public health education and communication, developed the interview guide (Appendix 1) using an iterative process. We created questions based on a literature review identifying key themes around clinical practices for adolescent and early HPV vaccination, in accordance with the study aim [18–21]. The guide was reiteratively developed in collaboration with a master’s-level Qualitative Research Specialist in implementation science and reviewed by a primary care provider with over 20 years of experience in family medicine and maternal and child health. The guide probed primary clinic personnel on the perceived importance of HPV vaccination, the messages used to recommend HPV vaccination, the willingness to promote early HPV vaccination, and the perceived facilitators and barriers to promoting early HPV vaccination.

Key informant interviews were conducted post hoc, after learning that they were already recommending HPV vaccination to their younger patients. The same interview guide that was used for primary clinic personnel interviews was used for key informant interviews, though some questions were modified to ask key informants specifically about their process for recommending HPV vaccination to 9- and 10-year-old patients, rather than their willingness to do so (Appendix 2). Key informants were also asked to assess the feasibility of implementing age-9 HPV vaccination on a larger scale, including in clinics with fewer human and technological resources.

### In-depth interviews

Thirty-minute interviews were conducted via Zoom by the study Principal Investigator (NAV) and a qualitative research assistant (RML) between April and July 2022. This study of provider practices and attitudes toward HPV vaccination was determined not to constitute human subjects research by the Institutional Review Board of the University of North Carolina at Chapel Hill (UNC) (IRB #: 21–0182). As such, written informed consent was not required, and interviewees were instead emailed a description of the study design ahead of time, informing them of the nature of the interview prompts and the fact that interviews would be digitally recorded. Interviewees verbally consented to audio and video recording at the start of each interview prior to initiating the recording and received $50 Amazon gift cards for completing the interview. Zoom’s auto-transcription feature was used to generate preliminary transcripts. The research assistant then reviewed and corrected auto-transcription errors for each interview to reflect the content of the interview based on audio and video recordings.

### Analysis

We used a rapid assessment approach to identify overarching patterns and used thematic content analysis to summarize key findings. To address the feasibility of age-9 recommendation of HPV vaccination in primary clinics, we identified and agreed upon an initial set of topical codes and interpretive codes using preliminary memos and discussion of themes. A codebook was developed to further define each code prior to coding all the transcripts. We used the online Dedoose software version 9.0.54 (SocioCultural Research Consultants, Los Angeles, CA) to apply topical codes to excerpts from qualitative transcripts. To address inter-rater reliability, two study staff (NAV, RML) independently coded two different transcripts. The frequency and consistency of code application among the study staff were compared using the Code Application feature in Dedoose and discussed between the study staff to increase consistency between coders and increase the internal validity of the study analysis. In the first two transcripts, the two study staff had 65.5% agreement in code use. After resolving coding discrepancies, we fine-tuned the codebook to indicate more specifically when to apply and not to apply each code. While this feasibility study did not intend to reach saturation of themes from a small number of interviews, we identified several themes that were reported by multiple interviewees and report these in the main results.

## Results

### Interviewee and clinic characteristics

We completed a total of 10 in-depth interviews with personnel from two primary clinics, including 2 providers, 4 nursing staff, 3 medical assistants, and 1 practice manager (Table 1). We also completed two in-depth interviews with key informants who were both medical providers in urban academic clinics; one provider was employed at a family medicine clinic, and the other was employed at a pediatrics clinic (Table 1).

**Table 1:** Characteristics of two North Carolina clinics that provide HPV vaccination to adolescents

	**Primary clinic A**	**Primary clinic B**
County	Chatham	Alamance
Rural residents in the county (*N*, [%])	41,864 (66) [22]	47,561 (28) [23]
Primary provider specialty	Family Medicine	Family Medicine, Pediatrics
Number of health care providers (number full-time)	18 (6)	8 (5)
Number of medical staff (nurses, medical assistants)	12	7
Clinic performs routine patient outreach for HPV vaccination	Yes	No
Clinic has standing orders for HPV vaccination	Yes	Yes
Clinic uses a script or guiding language for HPV recommendation	No	No
Active patients ages 9–14 years^a^	414	966
Active patients preferring care in a language other than English (*N*, [%])	277 (67)	622 (36)
Payer mix of active patients (*N*, [%])		
Medicaid	219 (53)	690 (71)
Health Choice/CHIP	33 (8)	12 (4)
Private	25 (6)	60 (6)
Uninsured	137 (33)	176 (18)
Active patients ages 9–14 with ≥ 1 HPV vaccine dose documented in electronic medical record (*N*, [%])		
Age 9	0 (0)	1 (1)
Age 10	0 (0)	7 (4)
Age 11	11 (21)	48 (35)
Age 12	54 (67)	122 (79)
Age 13	63 (79)	170 (85)
Age 14	68 (80)	120 (88)
Total	196 (47)	468 (48)
Active patients ages 9–14 with ≥ 2 HPV doses documented in electronic medical record (*N*, [%])		
Age 9	0 (0)	0 (0)
Age 10	0 (0)	4 (3)
Age 11	1 (2)	12 (9)
Age 12	10 (12)	49 (32)
Age 13	27 (34)	97 (48)
Age 14	43 (51)	87 (64)
Total	81 (20)	249 (26)
Patients who completed 2 HPV vaccine doses by age 13, by age at first dose (*N*, [%])^b^		
Age 9	1 (100)	36 (100)
Age 10	4 (100)	15 (100)
Age 11	109 (97)	296 (97)
Age 12	24 (71)	52 (69)
Age 13	1 (13)	2 (17)
Total	139 (87)	401 (89)

^a^Active patients include those with a well-child check in the last 18 months, as of the query date July 22, 2022. Current patient age reflects their age at the time of the query

^b^Among all patients in the age range 9–14 years between October 2020 and March 2022. Age represents age at receipt of first HPV vaccine dose and does not reflect current patient age

Primary clinic A was located in a county where most residents lived in rural areas, in contrast to primary clinic B (Table 1). Both clinics had a family medicine focus, though primary clinic B additionally employed pediatricians and served a larger pediatric population. Both clinics had standing orders for HPV vaccination, though only primary clinic A performed routine patient outreach to schedule vaccination appointments, in part because of a smaller clinical and support staff. Neither clinic used a script or specific language to recommend HPV vaccination. Primary clinic B, having a pediatrics focus, served over twice as many patients ages 9–13 as primary clinic A. Most patients in both clinics received Medicaid or other public insurance, and both clinics had a substantial proportion of uninsured patients.

As of July 22, 2022, both clinics had comparable proportions of active patients ages 9–14 years with at least one documented HPV vaccine dose (primary clinic A: 196 patients, 47%; primary clinic B: 468 patients, 48%), and similar distributions of HPV vaccination initiation and completion by current patient age (Table 1). At the time of the query, none of the current 9-year-old patients and only four of the current 10-year-old patients had initiated HPV vaccination, and up-to-date routine vaccination by age 13 only reached 12% in Primary clinic A and 20% in Primary clinic B (Table 1). However, rates of completion by 13 gradually decreased with older age at initiation (Table 1). In both clinics, 100% of patients who initiated vaccination at ages 9–10 completed the series by or at age 13, compared to 96% in Primary clinic A and 91% in Primary clinic B who initiated at ages 11–12; less than 20% who initiated at age 13 finished the vaccine series by the end of the 13th year (Table 1). EMR data were cross-checked against data from the North Carolina Immunization Registry to confirm that vaccinations were documented in both sources and that no vaccinations were missing from EMR.

### Thematic analysis—primary clinic personnel

We identified four predominant themes from the interviews: (1) clinics have created opportunities to recommend HPV vaccination during well-child visits; (2) providers educate caregivers who are hesitant about HPV vaccination; (3) providers often consider the benefits of HPV vaccination in the context of adolescent social and physical development; and (4) providers are generally willing and able to promote age-9 HPV vaccination in the clinic. Themes and illustrative quotations are summarized in Table 2.

**Table 2:** Major themes and illustrative quotations identified from in-depth interviews (*n* = 10)

*Area of inquiry*	*Theme*	*Quotations*
Current clinical approaches to adolescent HPV vaccination	Existing opportunities to recommend HPV vaccination	“Usually, we go by whatever NCIR is recommending, like the state of North Carolina registrations recommendation. And every time I pull a record for one of our patients, I just look and see if they have already gotten [vaccinations] or are due soon.” (Primary clinic A) “At any pediatric visit, we’ll print NCIRs, and so we try to pay attention at any visit to be able to offer [the HPV vaccination].” (Primary clinic B)
	Addressing caregiver concerns and continuing education	“And even if they still have questions, we get the provider… even if they’re with another patient, they go back into answer the parent’s questions.” (Primary clinic B) “I feel like some parents, they feel like we're administering the disease into their child.” (Primary clinic B)
Receptiveness to promoting HPV vaccination among 9- and 10-year-olds	Considering HPV vaccination and child social and physical development	**“**I just think the parents aren't ready to think that their kids are growing up.” (Primary clinic B) “Looking at my grandchildren, after five, they’re not really getting any shots. So, if they're still coming for the well-child check, go ahead and plant that seed [regarding HPV vaccination].” (Primary clinic A)
	Assessing provider feelings of early vaccination	“The vaccine works better the younger we give it.” (Primary clinic B) “I think age-9 is better… you just can't predict when like kid will have that first experience with someone. Some 12-year-olds are really far away from that, and other 12-year-olds are not, and so I think that if you wait, it's just risky.” (Key informant B) “I think if you have a year of hesitancy, having that year age-9 is going to make it more likely to stay on time.” (Key informant A)
	Willingness and ability to promote age-9 HPV vaccination in the clinic	“… It sort of fit neatly into a new schedule to do the Tdap and the HPV at 10, and then the HPV and the [MenACWY] at 11.” (Key informant A) “I can see where that would be really exciting to be part of [an age-9 vaccination pilot trial]…then we'd have those results, and we can say this is better.” (Primary clinic B)

#### Existing opportunities to recommend HPV vaccination

Interviewees at both clinics reported that HPV vaccination is standard of care for adolescents, and predominantly occurs during routine well-child visits. In preparation for well-child visits, a medical assistant (MA) will review the patient’s EMR to identify any gaps in vaccination according to the CDC’s recommended vaccine schedule. MAs also review the North Carolina Immunization Registry (NCIR), where use of state-purchased vaccines is required to be reported, to review patient eligibility for vaccination. Alerts are triggered in both systems at age 11 when a patient is eligible to receive adolescent vaccines per CDC recommendations.

Though not routine, vaccinations are occasionally offered during sick visits to take advantage of the opportunity:

There are times where kids come in for non-well-child checks and are due for vaccines.... sometimes people will be like well, it’s not a well-child check, so we’re not going to do vaccines. I’m like well, they’re here. (Primary clinic A, Clinic Staff)

In addition, both clinics offer “nursing only” visits in which patients can receive vaccines without a provider consultation. During typical well-child visits, however, while MAs and nurses were involved in rooming patients and providing an overview of which vaccines the patient is due for, more in-depth counseling on recommended vaccinations was within the purview of the provider. Clinic staff and providers alike reported that caregivers who expressed vaccine hesitancy when interacting with clinic staff were more likely to respond to vaccine recommendations from their child’s regular caregiver:

If the provider [is] still kind of reviewing the next patient’s chart, I’ll kind of just get them real quick, I’m like hey they have questions, concerns. Because some parents will say yes to the provider. (Primary clinic B, Clinic Staff)

#### HPV vaccination education for hesitant parents

Interviewees at both clinics reported that they rarely encountered hesitancy from caregivers to vaccinate their adolescents against HPV. However, interviewees had difficulty estimating their adolescent vaccination rates, and perceptions of the need to improve HPV vaccination practices differed even within clinics.

We have no idea. We see a lot of adolescents. (Primary clinic B, Provider)

I guess I have not had anyone that I remember that refused the HPV vaccination. (Primary clinic A, Provider)

We need to work on the HPV vaccination rate. (Primary clinic A, Clinic Staff).

When asked how they handled HPV vaccination hesitancy or questions from caregivers, all interviewees responded that they educate caregivers on the purpose of HPV vaccination and its safety profile. The most common messages include the vaccine’s role in preventing HPV-associated cancers later in life.

Mostly it’s in the 30s that we’re seeing positive [cervical cancer screenings] for women, and I saw one male have a positive, so they’re like your child can get this later in life. We can vaccinate now to prevent. (Primary clinic A, Provider)

[HPV is] a virus that causes really gross warts in your private area, but it can also lead to cancer later… we know that any person can get cancer in the future. (Primary clinic B, Clinic Staff)

At times, an MA will brief the caregiver and patient on vaccines that are due, and they will report to the provider if any hesitancy was expressed. Providers reported that they were often able to persuade a hesitant or questioning caregiver to accept HPV vaccination following a brief discussion of the benefits of the vaccine. Other times, caregivers have misconceptions about HPV vaccination including beliefs that HPV vaccination itself could cause cancer and that HPV and HPV-associated cancers do not affect males. Providers mentioned working actively to correct any misconceptions that might arise.

Well, I think in general the word “cancer” is pretty scary for people…I feel like some parents, with any vaccine, they feel like we’re administering the disease into their child. (Primary clinic B, Clinic Staff)

Whenever they raise concern(s) about gender, I’m like, this is not just for females, this is for males as well…males and females are going to get HPV if they’re not immunized…there’s sometimes they change their minds. (Primary clinic A, Clinic Staff)

#### HPV vaccination relates to social and physical development

The child’s age and developmental stage were common considerations among interviewees when discussing HPV vaccination with caregivers. Several reported that caregivers expressed that HPV vaccination was not appropriate for their adolescents, either because they are not yet sexually active and not at risk for HPV or because they perceived HPV vaccination as an enticement to initiate sexual activity. Several interviewees reported that they had children or grandchildren of their own and would want them to be protected against HPV-associated cancers.

I’ve certainly heard of people being hesitant to receive the vaccine because of concern regarding sexual promiscuity at a young age. (Primary clinic A, Clinic Staff)

I’m a mom. I have three kids, one of them is a male and two females. I really want them to be as protected as they can be. (Primary clinic A, Clinic Staff)

When asked for their opinions on the ideal age to initiate HPV vaccination, all interviewees reported that 11–12 years (*n* = 2) or younger (*n* = 7) was ideal. The common justification was that HPV vaccination should be given prior to the onset of sexual behavior to be most effective. Some interviewees reported that the youngest eligible age (e.g., 9 years) was ideal, as they had heard of or encountered adolescents who became sexually active before age 11.

#### Providers are willing to implement age-9 HPV vaccination

When asked if they would be willing to recommend HPV vaccination at ages 9–10 years, some interviewees themselves expressed hesitancy. Some believed that age 9 was too young and that age 10 or older was sufficiently early to initiate HPV vaccination.

... It does feel a little bit funny and maybe that’s because I have children in the single digits. I don’t want my child to be a teenager at 9… (Primary clinic A, Provider)

Ten and 11 go to where you’re going to middle school now and they are hearing more and more about it…9, I think it is too young. It’s only one year difference, but I don’t know, it sounds young. (Primary clinic B, Clinic Staff)

Some interviewees initially questioned the benefits of age-9 vaccination. When the interviewers provided some probes, interviewees tended to support age-9 vaccination*.* Interviewees also generally agreed that age-9 vaccination could be implemented in their practices with additional training and some minimal changes to current practices. However, some expressed reservations, suggesting that staff are not always amenable to change and that it could be difficult to convince all personnel to embrace age-9 HPV vaccination.

I will say more training and acknowledgment of this vaccine would be a really nice factor. Like reeducate staff on how to approach when it comes to offering a vaccine and using the word ‘highly recommended.’ (Primary clinic A, Clinic Staff)

I’ll be honest, at our site… change is hard here. (Primary clinic A, Clinic Staff).

### Thematic analysis—key informants

Two key informants, one physician in a pediatrics practice (key informant A) and one in a family practice (key informant B), reported beginning discussions about HPV vaccination with their 9- and 10-year-old patients. The pediatrician reported that the shift to vaccinating younger patients was a response to the clinic’s quality improvement (QI) metrics and that initiating discussions about HPV vaccination age-9 at ages 9 and 10 increased the number of patients who received on-time vaccination according to clinic records.

Other providers in the practice also adopted this strategy, in part to receive monetary incentives for having high metric scores. However, adoption was not universal, and a provider hesitated to support practice-wide mandates for early vaccination:

And so, I’ve been reluctant to have a practice-wide policy on timing [sic: age] about administration. I think it’s fine to have a practice-wide policy on promotion. (Key informant A, Provider)

This provider implemented an alternative vaccination schedule that retained the CDC recommendation to co-administration of HPV with Tdap and MenACWY vaccines but allowed for age-9 completion of the series, more time to complete the series on time, and fewer vaccinations per visit. When asked about challenges to implementing age-9 HPV vaccination across the practice, this provider mentioned very few barriers apart from having support from all providers. It was expressed that monitoring vaccination at ages 9–10 was easy as long as vaccinations were aligned with annual well-child visits:

That’s not hard... We try to do well visits on or after the birthday. With 6 to 12 months between doses one and two, if you just give it at your 10-year-old well-visit and then your 11-year-old well-visit, you’re fine, you don’t really need to track [the timing between doses]. (Key informant A, Provider)

A family medicine provider reported recommending age-9 vaccination in response to the possibility of early sexual initiation. They also reported using every clinical opportunity to encourage vaccination, usually in advance of the CDC-recommended timeline:

The more times you mentioned something, the more likely someone is to be comfortable with it and just get it done… I talk about vaccines at almost every visit for everybody.... (Key informant A, Provider)

In contrast to the pediatric practice, one interviewee noted a limitation to implementing an age-9 vaccination approach in a family medicine practice given the small number of adolescent patients and less urgency to adapt their existing systems.

It’s difficult because if you don’t have as many kids, then you don’t really have the structure to make it really easy for parents. (Key informant B, Provider)

This provider suggested age-9 vaccination could reasonably be incorporated into current practice and that modifications to existing EMR-based alert systems that provide notices when a patient is overdue for recommended vaccines would facilitate age-9 vaccination.

So, if it doesn’t add to the number of visits, then it seems to make sense to just do [HPV vaccination] at 9 and 10 and you can kind of get that over with, and then at 12, then they get their next you know set of vaccines... If they need time to think about it, then they have time to think about it. So, I feel like it makes sense to talk about it age-9 rather than later. (Key informant B, Provider)

I do think that, if the [EMR alerts] fired at age 9 and said… your patient is now due for HPV, it probably would make a difference... At least we give the vaccination information sheet at that visit and then they have it ahead of time. (Key informant B, Provider)

## Discussion

In interviews with providers and staff from clinics providing HPV vaccination to adolescents, we found a high level of support for age-9 HPV vaccination at ages 9 and 10 years of age. Interviewees in urban and rural clinics suggested that their clinic procedures could reasonably be adapted to start recommending vaccine age-9 than the CDC-recommended 11–12 years, with additional staff education or training needed. While EMR data from participating clinics show that fewer than half of patients had completed the HPV series by age 13, there is evidence that patients who initiated age-9 were more likely to have completed vaccination, and to have completed on time. According to interviews with key informants from urban academic settings, some providers are already successfully providing HPV vaccination age-9, and lessons learned can possibly be adapted to other primary care settings, including in rural areas, to improve the coverage of on-time HPV vaccination.

Because awareness of the HPV vaccine is such an influential factor for caregivers of adolescents, an important consideration of age-9 HPV vaccination recommendations is whether clinic personnel are aware that it can be administered to 9- and 10-year-olds [24]. Prior studies on the experiences of clinic personnel with age-9 HPV vaccination did not measure their initial awareness of this option, which might have implications for its perceived acceptability [14, 15, 25, 26]. In our study, two interviewees were not aware that 11 years was not the minimum age for HPV vaccination initiation, suggesting that they had not previously considered vaccinating younger patients and that they had no preconceived notions about the practice. Furthermore, even among interviewees who were aware of the FDA approval for 9- and 10-year-olds, several could not initially describe any benefits of age-9 HPV vaccination. Continuing education for providers and clinic staff should emphasize not only that HPV vaccination is indicated for younger patients, but also that emerging data suggest that it can lead to better on-time coverage and optimal prevention. Recent research suggests utilizing the theory of planned behavior to create provider communication interventions, such as announcement training instead of conversation training, to increase perceived behavioral control in providers, leading to a more effective recommendation and ultimately an increase in HPV vaccine series completion [27].

The two key informants from two academic clinics were in different stages of implementation of age-9 HPV vaccination: one had developed a protocol for early vaccination, including an alternative vaccine schedule, whereas the other simply introduced HPV vaccination age-9. These differing approaches could reflect differences in the perceived importance of tracking adolescent vaccination rates between pediatrics and family practices. Tracking patient vaccination rates using EMR or immunization registries is an evidence-based practice to motivate providers to vaccinate their patients in a timely manner [28–32], and only the pediatrician key informant emphasized QI metrics as a motivator to vaccinate age-9. In contrast, family practitioners tend to see fewer adolescent patients than pediatricians and are possibly less likely to report or fulfill QI measures related to adolescent vaccines, including HPV vaccination [33–35]. While this feasibility study cannot draw firm conclusions on the differences in motivations between pediatrics and family practices, more research on this topic is warranted.

Some practice-based challenges make it difficult to start recommending and administering HPV vaccination at ages 9–10. Interviewees reported relying on notifications from EMR or NCIR to determine when a patient was due for vaccination. While NCIR indicates an “earliest date” for vaccination at age 9, based on the patient’s recorded age, the “recommended date” is age 11 in accordance with CDC recommendations [36]. Changes to these recommended ages identified by these alert systems might impel staff and providers to recommend vaccination to 9- and 10-year-olds without making any additional efforts. This successful change has been documented in a prior study of QI measures in a primary care network based in Columbus, Ohio, and more information is needed to understand how software programming decisions and modifications can be made in different clinic systems [14].

This feasibility study included a small number of clinics, all of which were affiliated with or proximal to a large, well-resourced academic center, and do not represent the experiences of clinics in more remote areas and with fewer pediatric providers. Primary clinic B was located in a county that had a lower proportion of rural residents compared to primary clinic A (28% versus 66%), and the patient populations served by the two clinics might differ with respect to sociodemographic factors. However, this difference allows for an interesting comparison between the two clinic types in terms of perceptions of acceptability and feasibility of age-9 HPV vaccination. In addition, all participating clinics had sophisticated EMR that could be programmed to indicate when vaccines were due. One limitation of this data is that clinic EMR do not capture vaccinations that happened outside of the clinic system, and vaccination coverage based on EMR is likely underreported. Clinics with more rudimentary systems or paper records would have to identify other strategies for identifying vaccine-eligible 9- and 10-year-olds and monitoring vaccine receipt. However, the alternative vaccination timeline proposed by one of the key informants (i.e., providing HPV and Tdap vaccination at age 10, and HPV and MenACWY vaccination at age 11 during annual well-child visits) could be implemented in any primary care clinic, with no need for additional tracking or alerts.

HPV vaccination of 9- and 10-year-olds carries several advantages and could be facilitated with provider education and support. However, while modifications to vaccination schedules can be implemented without major disruptions to current practice, it can be difficult to build support for such changes in rural settings where adolescents face reduced access to primary health care, scarcity of pediatric specialty clinics with the infrastructure to promote adolescent vaccination and providers who actively recommend it, and higher prevalence of vaccine hesitancy compared to adolescents in urban settings [37–41]. Of note, primary clinic A, which served a mostly rural population, had nearly twice as many uninsured patients as primary clinic B, providing further evidence that children in rural areas face more barriers to health care in general and might have fewer opportunities to receive HPV vaccination. This exploratory qualitative study provides insights into how HPV vaccination is perceived by providers in real-world settings and allows the research team to find effective methods to promote age-9 HPV vaccination as an effective, convenient, and acceptable practice for patients in rural settings. Research on rural caregiver acceptability of age-9 HPV vaccination and implementation of age-9 HPV vaccination in rural clinics are planned, which will inform strategies to tailor this approach for optimal effectiveness in rural settings.

Globally, HPV vaccination programs suffer from suboptimal uptake, with only 6% of countries achieving at least 90% completion of the vaccine series, and with low- and middle-income countries (LMIC) performing worse than high-income countries [42]. Vaccination barriers in LMICs are similar to those that exist in the rural USA, including reduced health care use and access to vaccination services. School-based vaccination and younger age at vaccination are strategies more often used in LMICs, to reduce the burden of clinic visits by offering vaccination at school and capturing school-aged children before they drop out of school [42–44]. A similar strategy of leveraging existing opportunities for vaccination and offering vaccination at the earliest eligible age may also be appropriate for rural areas of the USA, and future pilot studies to assess the implementation of age-9 HPV vaccination in these settings will help to refine and maximize the potential impact of this intervention, leading to scale-up and large-scale evaluation of the intervention. Plans for more in-depth research on age-9 vaccination in rural NC are underway, and the methods and findings from these studies will be published and accessible to researchers and clinicians globally, who can further adapt them to specific contexts. This information can be particularly useful to LMIC that do not yet have HPV vaccination integrated into their national immunization strategies and would benefit from implementing evidence-based practices for HPV vaccination from the beginning. In late 2022, the World Health Organization recommended implementing single-dose HPV vaccination in resource-limited settings, given evidence for sustained immunogenicity and efficacy of a single dose of bivalent or nine-valent HPV vaccine [45]. In combination with age-9 vaccination, this new recommendation promises to further maximize the efficiency of HPV vaccination interventions globally, substantially reducing the risk of cervical cancer for the populations with the greatest barriers to preventive care.

## Conclusions

An age-9 vaccination practice could be piloted in a small number of clinics that are willing and able, and lessons learned could be used to make practice-specific improvements to age-9 vaccination practices. Findings from these studies could support effectiveness trials of a novel early HPV vaccination intervention and recommendations for implementing early vaccination in a variety of settings.

### Supplementary Information

**Additional file 1.** Interview guide for key informants.

**Additional file 2.** Interview guide for primary clinic participants.

## Data Availability

Data available on request from the authors.

## Abbreviations

CDC: US Centers for disease control and prevention
EMR: Electronic medical record
FDA: US food and drug administration
HPV: Human papillomavirus
MA: Medical assistant
MenACWY: Quadrivalent meningococcal conjugate vaccine
NC: North Carolina
NCIR: North Carolina immunization registry
QI: Quality improvement
Tdap: Tetanus-diphtheria-acellular pertussis vaccine
