# Supplementary material for: Acceptability and readiness to promote human papillomavirus vaccination at ages 9–10 years: a feasibility study among North Carolina clinics
Source: Pilot Feasibility Stud. 2023 Aug 31;9:153. doi: 10.1186/s40814-023-01379-y (PMC10470204; doi:10.1186/s40814-023-01379-y)
Supplement: Supplementary file 2 — Additional file 2. Interview guide for primary clinic participants. [file 40814_2023_1379_MOESM2_ESM.docx]

**Acceptability and readiness to promote human papillomavirus vaccination at ages 9-10 years:**

**A feasibility study among North Carolina clinics**

***Interview guide for primary clinic participants***

**Introduction & Consent**

**Length of Interview:** 30 minutes

**Background (3 minutes):**

**Greeting:** Hello, my name is___________. I am part of a research team from the University of North Carolina. Thank you for agreeing to participate in our study about the potential of offering HPV vaccination to 9- and 10-year-olds. Is this still a good time for you to chat?

**Purpose:** The purpose of this study is to better understand the benefits and challenges to increasing HPV vaccine uptake by offering vaccination to 9- and 10-year-olds at clinics in rural North Carolina. To provide background, the Advisory Committee on Immunization Practices (ACIP) recommends universal vaccination beginning at ages 11-12 years, however HPV vaccine can be given starting at 9 years of age per FDA licensure. We are conducting interviews with clinic personnel to explore their perspectives on offering HPV vaccination to 9- and 10-year-olds.

**Process and Confidentiality:**

Please know that there are no right or wrong answers to any of these interview questions. Occasionally, I may need to move ahead with the interview, but please let me know if you want to add anything or if you have any questions. Please let me know if there are any questions that you would prefer not to answer, and we can skip those questions and move on. Our main goal is to learn from you and ensure that you feel comfortable sharing your thoughts, opinions, and experiences. This interview will last about 30 minutes.

Before we begin, I would like to state that the conversation is being recorded to help us remember what is said during this discussion. This is a research study and all responses will be kept confidential. No identifying information about you will be shared in any reports that are published from this interview. I hope that you will feel free to speak openly. Do we have your permission to start recording?

Do you have any questions before we begin?

[BEGIN ZOOM RECORDING]

**Section I: Establish a Context for the Discussion to Follow (5 minutes)**

*[READ]:* *To begin, I’d like to start by talking a little bit about your work in the [add name of health clinic]*

1.1: **What is your current job title?**

1.2: **How long have you been in your current role?**

1.3: **What role do you play in HPV vaccination at this clinic?**

[Probes]:

Recommend HPV vaccine to patients/caregivers

Administer vaccinations

Schedule vaccinations

Monitor vaccination rates

Provide vaccine education

Other (describe)

1.4: **According to the CDC’s National Immunization Survey, the state of North Carolina has achieved 80.3% coverage of at least one dose of HPV vaccine among adolescents ages 13-17 years (**[**https://www.cdc.gov/vaccines/imz-managers/coverage/teenvaxview/data-reports/index.html**](https://www.cdc.gov/vaccines/imz-managers/coverage/teenvaxview/data-reports/index.html)**). According to your clinic records, [XX]% of active patients ages 9-14 have received at least one dose of HPV vaccine. What is your reaction to the current rate of HPV vaccination among adolescents at your clinic?**

[Probe]: Do you feel that this rate is appropriate, or does it need to increase? Why?

**Section II: How do clinics currently approach HPV vaccination? (10 minutes)**

*[READ]: Thank you for sharing that information with me. Now I’d like to learn more about how HPV vaccination occurs at your clinic for patients ages 9-14 years.*

2.1**: Do you or providers at your clinic use any type of script when recommending HPV vaccination?**

*[IF USES SCRIPT]:*

1. What do you think about this script? What works well? What could be improved?
2. Do you or other providers make modifications to the script when discussing HPV vaccination with patients and caregivers? If so, can you please tell me more about this?
    [If they do]: How do you or providers decide when to make modifications?

[Probes]:

- Based on flow of conversation?
- Based on experience from previous vaccine conversations?

*[IF NO SCRIPT]:*

- - - 1. What key messages do you think are helpful when recommending the HPV vaccination to patients and their caregivers?

[Probes]:

- vaccine effectiveness against cervical cancer
- vaccine safety

2.2: **Do patients or caregivers ever raise any concerns about the HPV vaccine? What are some of these concerns?**

[Probes]:

- Vaccination is painful
- Vaccination is unsafe
- Child is too young
- Child is not at risk for HPV

2.3**: What do you say to a patient or caregiver who raises these concerns?**

2.4: **How long does it usually take to give recommendations about HPV vaccination to patients and their caregivers?**

1. Are there any specific parts of these recommendations that require more time to discuss?
2. Do you feel you have sufficient time to have this conversation during a visit? Why or why not?

2.5: **Why do you feel the HPV vaccine is important to the health of preteens and teens*?***

2.6: **Your clinic manager has indicated that your standard of care is to offer HPV vaccine to kids starting at age [XX].**

- - 1. Do you think that this is the ideal age to receive the HPV vaccination?
    2. [If yes] Why do you think this is the ideal age?
    3. [If no] What age do you think would be best to start recommending the vaccine? Why?

**Section III: Receptiveness to promoting HPV vaccination among 9-10-year-olds (10 minutes)**

*[READ]: Thank you for sharing that information with me. Now, I would like to ask you some questions about providing HPV vaccination specifically to 9 to 10 year-olds. As background, while the FDA has approved HPV vaccination for children starting at age 9-years-old, recommendations about when to start HPV vaccination differ across organizations. Currently, the CDC recommends initiating the vaccine series at age 11-12, with discretional use at ages 9-10 years.*

3.1: **How would you feel about recommending the HPV vaccination to your 9 to 10 year old patients?**

1. [If need more detail] Can you tell me more about what makes you feel this way?
2. How do you think caregivers would feel about vaccinating their 9-and 10-year-olds?
3. How do you think providers and other personnel in your clinic would feel about providing HPV vaccination to 9 to 10 year-olds?

3.2: **How might your conversation with caregivers when recommending for a 9-10 year old differ from when recommending to an older child?** [KEY POINT]

[Probes]:

- How might the length of conversation differ?
- How might the messages you deliver differ?

3.3 **Can you think of any benefits to offering vaccine to 9-10yos?**

[Probes]:

- More time to complete the 2-dose series “on time”
- Possibly avoids discussion/concern about sexual risk
- It is more convenient to vaccinate younger children, as they are more engaged in health care
- Younger kids might more compliant with vaccination recommendations

3.4: **Do you foresee any challenges to providing HPV vaccination to 9-10-year-olds at your clinic?**

[If “yes”, continue to next question. If “no”, proceed to III]

1. Can you describe these challenges?
2. What might alleviate these challenges to make it easier to vaccinate 9-10 year-olds.

[Probes]:

- Additional staffing? For what roles/purposes?
- Change to current tracking system and appointment reminders?
- Additional training for clinic staff and providers? What type of training?

[If no]

1. What factors could make it feasible to provide HPV vaccination to 9-10 year-olds at your clinic?

[Probes]:

- Tracking System
- Staffing
- Training

**Section IV: Closing (2 minutes)**

*[READ]: We’re almost at the end of the interview. I’d like to take a few minutes to reflect on some of the things we’ve talked about today.* ***Tell me:***

4.1: **Do you think that providing HPV vaccination to 9 to 10 year-olds would improve uptake and completion in your clinic? Why or why not?**

4.2: **Let’s say there was a clinical trial to estimate the effectiveness of early vaccination (9-10 years) versus standard of care (11-12 years) in increasing vaccine initiation and completion. Your clinic would have to be willing to be randomized to early or standard vaccination and routinely monitor vaccination rates among your patients. Do you think your clinic would be a good candidate to participate in such a trial? Why or why not?**

[Probe]: What additional resources might your clinic need to carry out these tasks?

4.3: **Is there anything else that we did not cover that you would like to share with me?**

Thank you for taking the time to speak with me today. I know that your time is valuable and I appreciate your insight on this topic.

[END ZOOM RECORDING]
